# External validation of clinical prediction models: simulation-based sample size calculations were more reliable than rules-of-thumb

**DOI:** 10.1016/j.jclinepi.2021.02.011

**Published:** 2021-07

**Authors:** Kym I.E. Snell, Lucinda Archer, Joie Ensor, Laura J. Bonnett, Thomas P.A. Debray, Bob Phillips, Gary S. Collins, Richard D. Riley

**Affiliations:** a Centre for Prognosis Research, School of Medicine, Keele University, Keele, Staffordshire, United Kingdom; b Department of Biostatistics, University of Liverpool, Liverpool, United Kingdom; c Julius Center for Health Sciences and Primary Care, University Medical Center Utrecht, Utrecht University, Utrecht, The Netherlands; d Centre for Reviews and Dissemination, University of York, York, United Kingdom; e Centre for Statistics in Medicine, Nuffield Department of Orthopaedics, Rheumatology and Musculoskeletal Sciences, University of Oxford, Oxford, United Kingdom; f NIHR Oxford Biomedical Research Centre, John Radcliffe Hospital, Oxford, United Kingdom

**Keywords:** Sample size, External validation, Clinical prediction model, Calibration and discrimination, Net benefit, Simulation

## Abstract

**Introduction:**

Sample size “rules-of-thumb” for external validation of clinical prediction models suggest at least 100 events and 100 non-events. Such blanket guidance is imprecise, and not specific to the model or validation setting. We investigate factors affecting precision of model performance estimates upon external validation, and propose a more tailored sample size approach.

**Methods:**

Simulation of logistic regression prediction models to investigate factors associated with precision of performance estimates. Then, explanation and illustration of a simulation-based approach to calculate the minimum sample size required to precisely estimate a model's calibration, discrimination and clinical utility.

**Results:**

Precision is affected by the model's linear predictor (LP) distribution, in addition to number of events and total sample size. Sample sizes of 100 (or even 200) events and non-events can give imprecise estimates, especially for calibration. The simulation-based calculation accounts for the LP distribution and (mis)calibration in the validation sample. Application identifies 2430 required participants (531 events) for external validation of a deep vein thrombosis diagnostic model.

**Conclusion:**

Where researchers can anticipate the distribution of the model's LP (eg, based on development sample, or a pilot study), a simulation-based approach for calculating sample size for external validation offers more flexibility and reliability than rules-of-thumb.

## Highlights

• After a clinical prediction model is developed, it is usually necessary to undertake an external validation study that examines the model's performance in new data from the same or different population. External validation studies should have an appropriate sample size, in order to estimate model performance measures precisely for calibration, discrimination and clinical utility.
• Rules-of-thumb suggest at least 100 events and 100 nonevents. Such blanket guidance is imprecise, and not specific to the model or validation setting.
• Our works shows that precision of performance estimates is affected by the model's linear predictor (*LP*) distribution, in addition to number of events and total sample size. Furthermore, sample sizes of 100 (or even 200) events and non-events can give imprecise estimates, especially for calibration.
• Our new proposal uses a simulation-based sample size calculation, which accounts for the *LP* distribution and (mis)calibration in the validation sample, and calculates the sample size (and events) required conditional on these factors.
• The approach requires the researcher to specify the desired precision for each performance measure of interest (calibration, discrimination, net benefit, etc), the model's anticipated *LP* distribution in the validation population, and whether or not the model is well calibrated. Guidance for how to specify these values is given, and R and Stata code is provided.

**What is new?**

**Key findings**
• Existing rules-of-thumb, such as having 100 events and 100 non-events, for the sample size required for external validation studies for prediction models of binary outcomes may not ensure precise performance estimates, particularly for calibration measures.
• Precision of performance estimates is affected by the model's linear predictor distribution, in addition to the number of events and total sample size.

**What this adds to what is known**
• Our simulation study shows that more than 200 events and non-events are often needed to achieve precise estimates of calibration, and the actual sample size calculation should be tailored to the setting and model of interest.
• Our new proposal uses a simulation-based sample size calculation, which accounts for the linear predictor distribution and (mis)calibration in the validation sample, and calculates the sample size (and events) required conditional on these factors.

**What is the implication, what should change now**
• Precise performance estimates should be targeted when externally validating prediction models for binary outcomes and this can be done through simulation. The approach requires the researcher to specify the desired precision for each performance measure of interest (calibration, discrimination, net benefit, etc), the model's anticipated *linear predictor* distribution in the validation population, and whether or not the model is expected to be well calibrated.

Alt-text: Unlabelled box

## 1 Introduction

Clinical prediction models utilise multiple variables (predictors) in combination to predict an individual patient's risk of a clinical outcome [1], [2], [3]. An important part of prediction model research is assessing the predictive performance of a model, in terms of whether the model's predicted risks: (i) discriminate between individuals that have the outcome and those that do not, and (ii) calibrate closely with observed risks (ie, predicted risks are accurate). This can be done by internal validation (such as bootstrapping) using the development data, and by external validation using independent data (ie, data different to that used for model development). Examining clinical utility (eg, a model's net benefit) is also important if the model is to be used to change (eg, treatment) strategies in clinical practice when predicted risks are above a particular threshold [4], [5], [6].

In contrast to model development studies [7], [8], [9], [10], relatively little research has been published on the sample size needed to externally validate a prediction model. For a binary outcome, often the number of events is used as the effective sample size [2], and therefore larger sample sizes are needed in settings where the outcome is rare. Steyerberg suggests having at least 100 events and 100 non-events for statistical tests to have ‘reasonable power’ in an external sample, but preferably >250 events and >250 non-events to have power to detect small but still important invalidity [11]. Other simulation and resampling studies conducted by Vergouwe et al. [12], Collins et al. [13], and van Calster et al. [14], also suggest having at least 100 events and 100 non-events to ensure accurate and precise estimates of performance measures, and even larger sample sizes (a minimum of 200 events and 200 non-events) to derive flexible calibration curves [13,14].

In this article, we evaluate whether the rule-of-thumb of having at least 100 (or 200) events and non-events is adequate for external validation of a prediction model with a binary outcome. A simulation study is used to investigate the relationship between various factors and precision of performance measures. Based on this, we suggest that sample size needs to be tailored to the setting of interest and propose a more flexible simulation-based approach to do this. Section 2 introduces predictive performance measures and describes the methods used for the simulation study and our simulation-based sample size calculation. Section 3 gives the results and the sample size approach is illustrated for validation of a prediction model for deep vein thrombosis (DVT). Finally, Section 4 provides some discussion.

## 2 Methods

### 2.1 Predictive performance measures and a motivating example

Consider a prediction model, developed using logistic regression for a binary outcome, that is to be externally validated. It will take the form,

(1) $$\begin{array}{ccc} \text{log}\left(\frac{p_{i}}{1-p_{i}}\right) & = & \alpha +\beta_{1}X_{1i}+\beta_{2}X_{2i}\\ & & +\;\beta_{3}X_{3i}+\;\cdots +\beta_{k}X_{\mathrm{ki}} \end{array}$$

where $p_{i}$ is the predicted probability of the outcome for individual *i, α* is the intercept, and the *X* and *β* terms represent the observed predictor values and predictor effects (log odds ratios) respectively. The right-hand side of the equation is often referred to as the linear predictor (*LP*). The predictive performance of a model is usually evaluated by estimating measures of calibration, discrimination and clinical utility, as defined in Box 1.

**Box 1:** Summary of typical performance measures to be estimated in an external validation of a logistic regression prediction model

**Calibration** • To estimate calibration performance of a prediction model, a calibration model can be fitted using the validation dataset. For a binary outcome, the typical calibration model is (2) $$\;\text{logit}\left(p_{i}\right)=\gamma +S\left(LP_{i}\right)\;$$ where $LP_{i}$ is the linear predictor value for participant i in the validation study as calculated from the existing prediction model (e.g. the right-hand-side of Eq. 1). • The calibration model can be used to obtain the calibration slope and the calibration-in-the-large: ○ The *S* coefficient is the estimate of the calibration slope and ideally should be 1. Values <1 indicate predictions are too extreme, for example low predicted probabilities are too low and high predicted probabilities are too high. Conversely, values >1 indicate that predictions are too narrow, for example low predictions are not low enough and high predictions are not high enough. *S* is typically below 1 for prediction models that are overfitted to the development data. ○ The calibration-in-the-large is estimated as *γ* when *S* = 1 (obtained by fitting Eq. 2 with *LP* included as an offset). This is closely related to the ratio of observed and expected outcomes (O/E), which is the average of the observed outcomes divided by the average predicted probability across all individuals. Estimates for *γ* should be equal to 0 if the model yields predictions that are perfectly calibrated at the population level. • A calibration plot is also essential to visually demonstrate the range of predicted risks, and their calibration with observed risks, ideally using a flexible (e.g. loess smoothed) calibration curve [14,15] The integrated calibration index (ICI) can be calculated to quantify the difference between the smoothed calibration curve and the ideal 45 degree line [16]. A similar measure is the estimated calibration index (ECI) [14]. **Discrimination** • Discrimination is assessed through the C-statistic, which for a binary outcome is equivalent to the area under the receiver operating characteristic curve. Values typically range from 0.5 for a model that discriminates no better than chance alone, through to 1 which would represent perfect discrimination. **Net benefit** • The overall consequences of using a prediction model for clinical decisions can be measured using the net benefit, [4,6] which expresses the relative value of benefits and harms associated with using the model to determine clinical decisions. Net benefit ($NB_{p_{t}})$ is $\;NB_{p_{t}}=\left(\text{sensitivity}\times \text{prevalence}\right)-\left(\left(1-\text{specificity}\right)\times \left(1-\text{prevalence}\right)\times \frac{p_{t}}{1-p_{t}}\right)$where sensitivity and specificity of the model predictions depend on the chosen risk threshold value $p_{t}$ for which clinical decisions are deemed necessary.	

### 2.2 Simulation study to investigate factors that influence the precision of performance estimates

We hypothesized that four factors relating to the external validation sample could affect the precision of performance estimates: (i) the outcome proportion, (ii) the total sample size, (iii) the standard deviation of the *LP* values, and (iv) the true (mis)calibration of the model. We conducted a simulation study to investigate this, as now described.

#### 2.2.1 Scenarios for the simulation study

We assumed the prediction model (which is to be external validated) has a *LP* that is normally distributed; *LP_i_* ~ Normal(*μ, σ*^2^). Scenarios for the simulations were defined using different values of *σ (*standard deviation of *LP*) and *μ* (mean of *LP*), as given in Table 1. The value of *μ* was selected to correspond to a particular “base probability” (*p* = inverse logit(*μ*) = 1/(1+exp(-*μ*)). This is the outcome event probability for an individual who has the mean *LP* value; alternatively, it can be considered the expected probability of an event in a population where *σ* = 0 and so *LP* is *μ* for all participants. When *σ* = 0, the base probability would be equal to the incidence (for prognostic studies) or prevalence (for diagnostic studies).

**Fig. 1:**
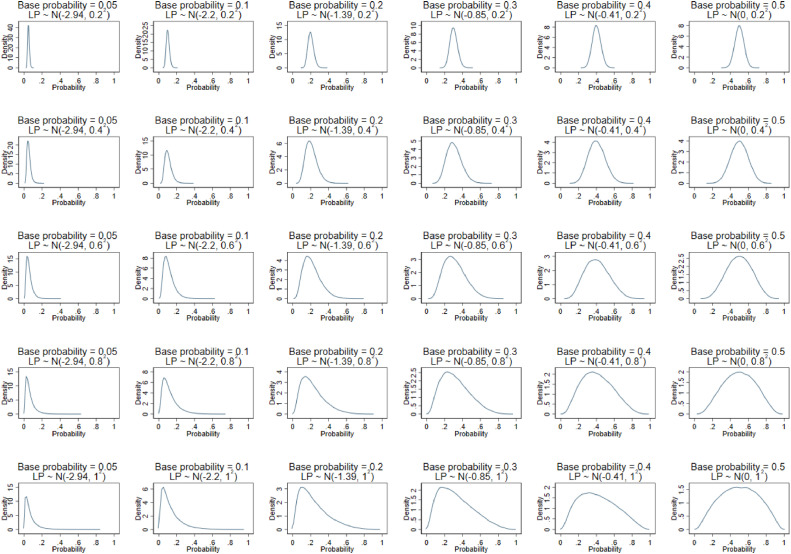
Distribution of the predicted probabilities for each of the different simulation scenarios based on the combination of base probability and σ values shown in Table 1.

We selected base probabilities to cover a wide range, from rare outcomes (~0.05) to common outcomes (0.5). Values for *σ* were chosen to provide a narrow through to a wide range of predicted probabilities from the model (depending on the outcome event proportion), as shown in Fig. 1. This also reflects low through to high values of the C-statistic, as the C-statistic will increase with wider distributions of *LP*. C-statistic values covered by the scenarios ranged from 0.56 when σ=0.2 to 0.75 when σ=1.0 and base probability=0.05.

#### 2.2.2 Main simulation process

The steps for the main simulation were as follows:

1) Define the simulation scenario by specifying *σ* and *μ*, with the latter corresponding to the “base probability” of the outcome (*p* = 1/(1+exp(-*μ*)). Also specify the desired expected number of events (*E*) in a population where all individuals have the base probability.
2) Set the validation dataset's sample size (*N*) using *E* divided by the base probability.
3) Generate *LP* values for each patient in the dataset using *LP_i_* ~ Normal(*μ, σ*^2^).
4) Generate binary outcomes (*Y_i_* = 0 or 1*)* for patient's by *Y_i_* ~ Bernoulli( 1/(1+exp(-*LP_i_*))).
5) Estimate with 95% confidence intervals (CIs) the model's calibration and discrimination performance using the external validation dataset.
6) Repeat steps 2 - 4 a total of 500 times for each simulation scenario. 500 repetitions was used to ensure a small Monte Carlo error whilst ensuring computation time was acceptable.
7) For each performance measure, calculate the average estimate and the average precision (based on the average 95% CI width) the 500 results.

For step 5, we estimated the calibration slope, calibration-in-the-large, the C-statistic and E/O statistic. Standard 95% CIs were calculated on the original scale (ie, estimate ± 1.96 x standard error) for all measures except the E/O statistic, which was derived on the log scale and then back transformed [17]. Using the simulation results for the various scenarios, we examined what factors were associated with increased precision of performance estimates. We also examined whether the precision was adequate when the sample size met the rule-of-thumb of 100 (or 200) events.

#### 2.2.3 Extensions to miscalibration

Step 4 assumes that the prediction model's *LP* is correct, such that the true calibration model is perfect (ie, intercept and slope are 0 and 1, respectively, in Eq. 2). Therefore, the scenarios were also extended to assess the effect of miscalibration. To do so, steps 1-3 remained the same but then we also created, *LP_miscal_* in which the original *LP* was multiplied by a ‘miscalibration factor’ (values of 0.80, 0.85, 0.90, 0.95, 1.05, 1.10, 1.15, and 1.20 were considered). The true outcome values in step 4 were then based on *LP_miscal_* rather than the original *LP*. Hence, in step 5 model performance estimates reflected a miscalibrated model; in particular, true calibration slopes were not 1.

### 2.3 Proposal for simulation-based sample size calculations

Rather than using a rule-of-thumb, we propose a simulation-based approach to identify the sample size required to achieve precise performance estimates. The proposal follows similar steps to that described previously for our simulation study, except now the process is iterative and converges when the minimum sample size is achieved. It is summarized in Box 2, and requires the researcher to specify the desired precision for each performance measure of interest (calibration, discrimination, net benefit, etc), the model's anticipated *LP* distribution in the validation population, and whether or not the model is well calibrated (ie, the values of parameters *γ* and *S* of the calibration model in Eq. 2).

**Box 2:** Steps of a simulation-based approach to calculate the sample size required for external validation of a particular prediction model for a binary outcome

1) **Specify the anticipated distribution of the prediction model's linear predictor (*LP)* in the validation study (eg, *LP_i_* ~ Normal(*μ, σ*^2^))** This might be based on the distribution reported for the development sample if the validation population is similar, or based on a pilot study if differences in case-mix are expected between the development and validation settings. 2) **Specify values for the parameters *γ* and *S* of the calibration model (**Eq. 2).* If development and validation populations are similar, a sensible starting point is to assume the model is well calibrated on average, ie, *γ* = 0 and *S* = 1. 3) **Specify the target precision for each performance measure.** For example, a 95% confidence interval (CI) width for the C-statistic < 0.1, 95% CI width for the calibration slope < 0.2, etc. 4) **Specify a starting sample size of the validation study.** For example, starting with N=100. 5) **Generate *LP* and true outcomes values for each participant.** Randomly generate the *LP* value for each participant using the distribution in step 1. Then, calculate the logit(*p_i_*) value for each participant using the calibration model specified in step 2. Then, randomly generate the true binary outcome *Y_i_ ~* Bernoulli(1/(1+exp(*-p_i_*)))*.* 6) **Calculate performance measures of interest for the prediction model in the external validation dataset and store estimates and 95% CIs.** For example, by comparing the model's predicted outcome risk outcome risk and the true outcome value for all participants in the dataset, estimate the C-statistic, calibration slope, calibration-in-the-large, E/O statistic, and net benefit (at particular risk thresholds). 7) **Repeat steps 5 and 6 for a specified number of repetitions.** For example, 500 repetitions. 8) **Using the stored estimates to calculate estimates of precision for each performance measure.** For example, the mean 95% CI width across the repetitions can be stored as the estimate of precision. 9) **Adjust the sample size and repeat steps 5-8 until the minimum sample size is identified that achieves the target precision for all performance measures.** *The process can also be repeated assuming different levels of miscalibration by altering the values of *γ* and *S* in Step 2, to see how this would affect sample size and the precision of estimates. For example, if the outcome event proportion is expected to be different in the validation sample than the development dataset, it is possible to adjust *γ* to achieve this new event proportion. Also, *S* < 1 might be assumed if the original model was overfitted and not corrected for optimism. See examples in 3.2.

A sensible starting point is to assume the model is well calibrated (ie, *γ* = 0 and *S* = 1) and that the *LP* distribution is the same as that for the development study, especially if the validation population is similar to the development population. The *LP* distribution may be obtained directly from the development study's publication or authors; if unavailable, it can be calculated indirectly using other information, such as the reported C-statistic or the distribution for each outcome group (eg, displayed at the bottom of a calibration plot) [17], [18], [19]. If the validation population is considered different from the development population (eg, due to a change in expected outcome proportion and/or case-mix), a pilot study may be necessary to gauge the distribution better. Further advice is given in the Supplementary Material.

The required precision is subjective and may be different for each measure. It helps to consider what width of a 95% CI is desirable for making strong inferences, and this may be context specific, especially for measures such as O/E and calibration-in-the-large (Supplementary Material). Our examples make some suggestions.

#### 2.3.1 Applied example: Diagnostic model for deep vein thrombosis

Debray et al. developed a diagnostic model for deep vein thrombosis (DVT) using data from 1295 individuals with about 22% truly having the outcome [20]. The model contained eight predictors, and overfitting was not a major concern given a large number of events per predictor. The model's linear predictor distribution was reported for their development cohort and also other settings, and we use this to illustrate our simulation-based approach for calculating the sample size for an external validation study of the DVT model. Example code is given in the supplementary material for Stata and is available on github for R (https://github.com/gscollins1973/External-validation-sample-size).

## 3 Results

### 3.1 Factors associated with the precision of model performance estimates: results from simulation study

#### 3.1.1 Precision of the estimated C-statistic

**Fig. 2:**
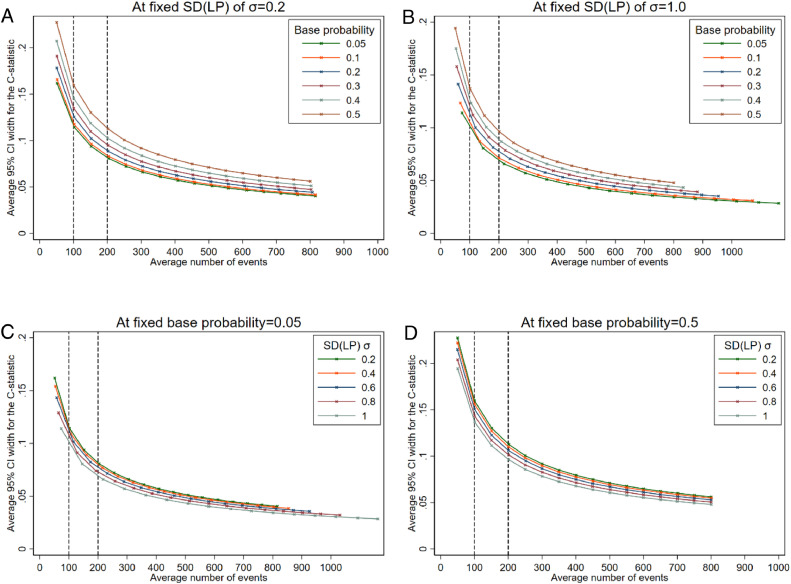
Average 95% confidence interval width for the C-statistic at different effective sample sizes (based on average number of events in the simulation scenario) comparing by base probabilities at fixed SD(*LP*) (panels A and B), or comparing by SD(*LP*) at fixed base probabilities (panels C and D).

The simulation scenarios (Table 1, Fig. 1) represented models with C-statistics from 0.56 (when σ=0.2) through to 0.75 (when σ=1.0). Fig. 2 (Panels A & B) show that estimates of the C-statistic were more precise (based on the average 95% CI width) when the outcome was rare compared to a more common outcome, for a particular average number of events. This is likely because the total sample size needs to be much larger for a rare outcome to achieve the same number of expected events compared to a more common outcome. The standard deviation (σ) of the *LP* also affected the precision of the C-statistic (Fig. 2, Panels C & D). The estimates were more precise when σ was larger, although the difference in the width of the 95% CI for the C-statistic when σ=1 compared to when σ=0.2 was only between 0.02 and 0.06 (depending on base probability) even for studies with 50 expected events. As precision increased with increasing SD(*LP*) for the scenarios considered, we would therefore expect even larger C-statistics (eg, >0.8) than considered here to be even more precise.

**Table 1:** Factors varied in the simulation study to define scenarios

Factor	Values
Standard deviation of the *LP_i_* (*σ*)	0.2, 0.4, 0.6, 0.8, 1.0
Base probability (inverse logit(*μ*))	0.05, 0.1, 0.2, 0.3, 0.4, 0.5
Expected number of events (*E*)	50, 100, 150, 200, …, 800

If an outcome was common (base probability=0.5) and σ=1.0, the average 95% CI widths were 0.14 and 0.09 with 100 and 200 expected outcome events and non-events, respectively (N=200 and N=400, respectively), (as seen in Fig. 2, Panel B or D). Therefore, with 200 events, a typical 95% CI would range from about 0.69 to 0.78. If we wanted a more precise estimate, say with a 95% CI width of 0.05, we would need at least 700 events (N=1400).

#### 3.1.2 Precision of the estimated calibration slope

**Fig. 3:**
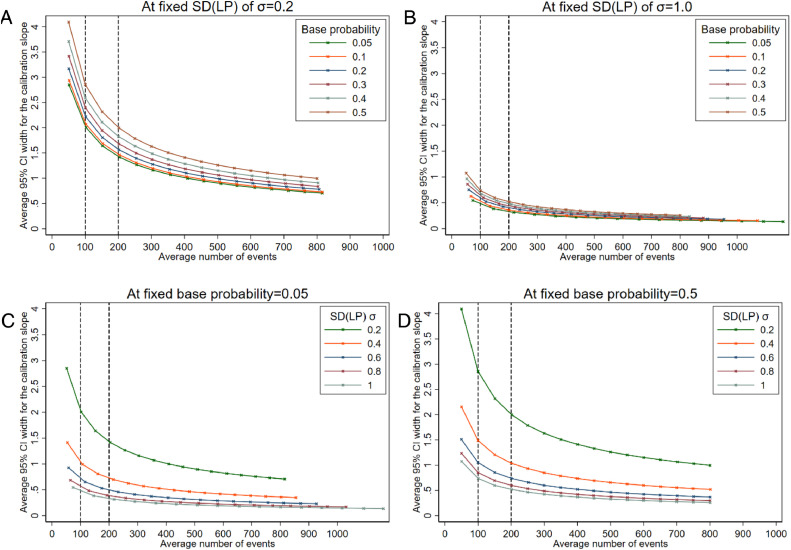
Average 95% confidence interval width for the calibration slope at different effective sample sizes (based on number of events) comparing by base probabilities at fixed SD(*LP*) (panels A and B), or comparing by SD(*LP*) at fixed base probabilities (panels C and D).

Estimates of the calibration slope can be very imprecise when the number of events is low. For example, in Fig. 3, across all scenarios the average 95% CI width is greater than 0.5 when there are around 50 outcome events, but can still be wide for studies with 100 or even 200 events when the outcome event proportion is high or *σ* is small. As seen in Fig. 3 (panels C & D) when *σ*=0.2, the average width of the 95% CI for the calibration slope is > 1 even for large studies with approximately 500 events on average. Although not as dramatic, estimates also become less precise as the base probability (and therefore the outcome event proportion) moves towards 0.5 (Fig. 3, panels A & B). Again, this is likely to be related to the difference in total sample size required to achieve the same number of events when the base probability differs.

If we wanted the average 95% CI width to be very precise, say a width of 0.2, we would need at least 400 outcome events if the outcome was rare (base probability=0.05) and the spread of the *LP* was large (*σ*=1.0). If we aimed for a 95% CI width of 0.4 (eg, 95% CI: 0.8 to 1.2), this would be achievable with 100 outcome events when the outcome was rare (base probability=0.05) and σ=1.0, but would require more than 300 outcome events if the outcome was more common (base probability>0.4 | *σ*=1.0) or if the distribution of *LP* was narrower (*σ*<1.0 | base probability=0.05).

#### 3.1.3 Precision of the estimated calibration-in-the-large and O/E statistic

**Fig. 4:**
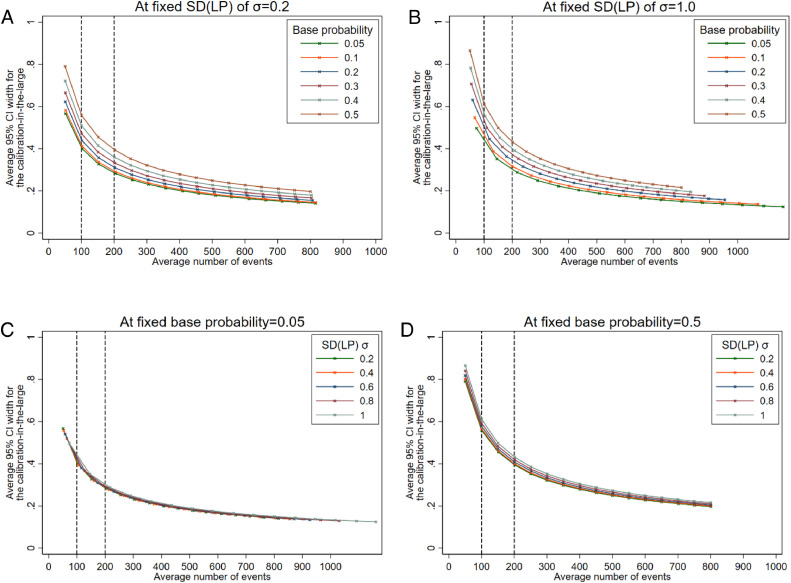
Average 95% confidence interval width for the calibration-in-the-large at different effective sample sizes (based on number of events) comparing by base probabilities at fixed SD(*LP*) (panels A and B), or comparing by SD(*LP*) at fixed base probabilities (panels C and D).

The 95% CIs for calibration-in-the-large were wide for low numbers of events, which indicates that in many circumstances 100 events is unlikely to be enough to obtain precise estimates (eg, a 95% CI width > 0.4). The standard deviation of the *LP* did not affect the precision much (Fig. 4, panels C & D). However, differences were seen for different base probabilities (Fig. 4, panels A & B). Findings for the ratio between observed and expected outcomes (O/E) were similar to those observed for calibration-in-the-large (Supplementary Figure S1).

#### 3.1.4 Extensions to scenarios with miscalibration

For the scenarios with miscalibration, each model was evaluated in different datasets (in which the model would be miscalibrated by varying degrees, as specified in Section 2.2.3). The precision in performance estimates was not greatly affected by miscalibration when the average number of observed events was still similar to that expected upon validation. However, performance estimates were less precise when miscalibration resulted in fewer events observed than expected. Supplementary Table S2 gives an example.

### 3.2 Application of simulation-based sample size calculation to go beyond current rules-of-thumb

The simulation study confirms that the precision in estimates of a model's predictive performance are affected by the standard deviation of the *LP* (σ), the outcome proportion (overall outcome risk), the number of events, and the total sample size. In contrast, adhering to blanket rules-of-thumb (eg, using 100 events) ignores these intricacies and fails to give precise performance estimates in some settings.

In contrast, our simulation-based approach to sample size calculation can be tailored to the model and population at hand (Section 2.3). That is, if researchers can specify the likely distribution of the model's *LP* and the outcome proportion in the target population, they can then use the simulation-based approach to identify a suitable sample size to ensure predictive performance estimates are precise.

To illustrate this, consider external validation of Debray et al's diagnostic prediction model for DVT (introduced in Section 2.3.1) [20], and the required sample size if:

a. the model is validated in the same population as the development cohort, and the model is expected to be well calibrated (*γ* = 0 and *S* = 1 in Eq. 2).
b. the model is validated in same population as the development cohort, but the model is expected to be miscalibrated (eg, due to overfitting) (*γ* = 0 and *S* = 0.9 in Eq. 2).
c. The outcome event proportion differs from the development data, either due to different case-mix or miscalibration of the model.

We consider these in turn, and compare to the rule-of-thumb of 100 or 200 events.

#### 3.2.1 Validation in the same population with good calibration

Debray et al. reported that in the development cohort the model's *LP* followed an approximate Normal(-1.75, 1.47^2^) distribution [20]. Assuming the external validation study has the same distribution, and that the model is well calibrated (*γ* = 0 and *S* = 1 in Eq. 2), we conducted simulations of external validation studies that have an average of 100 or 200 events. Table 2 shows the mean of the 95% CI widths for a range of calibration, discrimination and clinical utility measures. The 95% CI is fairly narrow for the C-statistic even when there are 100 events (mean width 0.09); it is also narrow for the integrated calibration index and net benefit (at an arbitrary clinical risk threshold of 0.1 for illustration). However, calibration-in-the-large and calibration slope estimates are imprecise with 100 events (eg, mean CI width 0.46 for slope), and even with 200 events (eg, mean CI width 0.33 for slope).

**Table 2:** Mean estimates and average 95% CI widths of performance estimates from 1,000 external validation studies with an average of 100 or 200 events, for validating the performance of a diagnostic model for DVT with an assumed linear predictor that follows a Normal(-1.75, 1.47^2^) distribution that is well-calibrated (*γ* = 0 and *S* = 1 in Eq. 2)

Performance Measure	N = 461 (~100 events on average in each validation dataset)		N = 922 (~ 200 events on average in each validation dataset)	
	Mean of the 1,000 estimates	Average width of 1,000 95% CIs	Mean of the 1,000 estimates	Average width of the 1,000 95% CIs
C-statistic	0.817	0.09	0.816	0.06
Calibration slope	1.016	0.46	1.008	0.33
Observed/expected	1.000	0.35	1.002	0.25
Integrated calibration index	0.020	0.04	0.014	0.03
Net benefit at a risk threshold of 0.1	0.153	0.08	0.154	0.06

Using the simulation-based process described in Box 2, we calculated the minimum sample sizes need to obtain average 95% CI widths of 0.1, 0.2, and 0.2 for the C-statistic, calibration slope, and ln(O/E), respectively (Table 3). This corresponds to an expected 95% CI of about 0.77 to 0.87 for the C-statistic, 0.9 to 1.1 for the calibration slope, and 0.9 to 1.1 for O/E, which we deemed precise for making strong inferences. We focus on the precision of O/E rather than calibration-in-the-large as it is easier to interpret. The results suggest that a sample size of 2430 participants (531 outcome events) is required, which is driven by the sample size required to estimate the calibration slope precisely. Clearly, if calibration is considered less relevant to, say, net benefit then a lower number may be sufficient for this particular model, given the narrow 95% CI width for net benefit even with 100 events (Table 2). However, calibration is an under-appreciated measure, and indeed linked to net-benefit [5], so we recommend it is nearly always important to assess.

**Table 3:** Sample size and number of events required to target precise performance measures in an external validation study of a DVT prediction model, with an assumed linear predictor that follows a Normal(-1.75, 1.47^2^) distribution and assuming the model is well calibrated (*γ* = 0 and *S* = 1 in Eq. 2).

Performance Measure	Targeted 95% CI width	Sample size (events) required to achieve CI width
C-statistic	0.1	385 (85)
Calibration slope	0.2	2430 (531)
Ln(observed/expected)	0.2	1379 (302)

#### 3.2.2 Validation in the same population but assuming miscalibration

Now we assume that in the validation population the model has the same *LP* distribution as in the development sample (Normal(-1.75, 1.47^2^)), but that the true calibration slope (*S* in Eq. 2) is 0.9 (eg, due to slight overfitting that was unaccounted for during model development) and *γ* is a non-zero value that ensures the outcome proportion is still 0.22 in the population. Aiming for the same CI widths as in the previous example, our simulation-based calculation now identifies the sample size required is 2141 participants (471 outcome events), again driven by the calibration slope. When the true calibration slope is assumed 0.8, the required sample size is lower still (1900 participants, 416 outcome events). Hence, the required sample size is lower the larger the miscalibration assumed.

#### 3.2.3 Validation in a different population with a different case-mix or event proportion

Lastly, consider a very different population from the development dataset, as shown by Debray et al. [20], where the outcome proportion is lower at 0.13 and the prediction model's *LP* distribution has changed (Normal(-2.67, 1.56^2^)), due to a different case-mix. Assuming the model is well calibrated in terms of the slope (*S* = 1 in Eq. 2), but setting the *γ* parameter value to a non-zero value so that the outcome event proportion of 0.13 is achieved in the population, our simulation-based approach identifies that 3156 participants (and 400 outcome events) are required, again driven by ensuring precise estimation of the calibration slope. This is substantially more than the 100 or 200 outcome events rule-of-thumb.

## 4 Discussion

Sample size for external validation studies should ensure precise estimates of performance measures of interest (eg, calibration, discrimination, clinical utility). Our simulation study shows that rules-of-thumb such as requiring a minimum of 100 events and 100 non-events (or even 200) do not give precise estimates in all scenarios, especially where calibration is of interest. Further, the precision of the C-statistic, calibration slope and calibration-in-the-large depends not only on the number of expected events, but also on the event proportion and therefore the overall sample size, as well as the distribution of the *LP*. Our proposed simulation-based approach accounts for these aspects, and is thus more flexible and reliable. Our examples illustrate how it calculates the required sample size for the particular model and validation setting of interest, and allows situations assuming calibration or miscalibration to be examined.

The sample sizes based on precision of performance statistics generally result in larger sample sizes than the rules-of-thumb, especially where calibration is of interest, in particular to estimate calibration slope precisely as demonstrated in our applied example (where 531 outcome events were deemed necessary) and the simulation study (eg, see Fig. 3 and Section 3.2.2). This contrasts work by others which showed that fewer than 100 events were required in some cases for validation of scoring systems based on logistic regression [21]. However, their calculations were based on achieving smooth calibration plots rather than ensuring precise estimates of numerically quantifying calibration. Applied examples also show imprecise estimates even when there are more than 100 events. For example, external validation of a prediction model for adverse outcomes in pre-eclampsia used a dataset with 185 events, and yet the 95% CIs for the C-statistic (0.64 to 0.86) and the calibration slope were wide (0.48 to 1.32) [22].

Our proposal to base sample size on precision of performance estimates is in line with Jinks et al., who suggest precisely estimating Royston's D statistic for survival prediction models. [23] Our simulation approach is more generalizable, as it can assess multiple performance measures simultaneously, and can be adapted for any outcome data type (eg, continuous, binary or survival). For survival data, simulations would also need to specify the censoring mechanism and key time-points of interest.

We focused on precise estimates of calibration, discrimination and clinical utility. Although the researcher should define the measures of key interest, generally we recommend that all are important to consider. Calibration and clinical utility, in particular, are often under-appreciated [24], [25], [26]. By ensuring precise estimates of calibration in terms of O/E (or the calibration-in-the-large) and calibration slope, this will help construct a reliable calibration plot. However, precise estimates across the entire range of predictions (eg, within each tenth of predicted risk from 0 to 1), would likely require even larger sample sizes. The simulation-based approach could also be extended to determine the sample size required to directly compare models, but again larger sample sizes are likely. If an external dataset is already available (ie, sample size is fixed), the approach can be used to ascertain the expected precision for that particular sample size and observed linear predictor distribution (to help justify its suitability).

We assumed that the linear predictor is normally distributed, which is supported by empirical evidence in some areas [27,28]. However, the simulation-based sample size approach (Box 2) can easily be adapted to use other distributions for the *LP*, as appropriate. If the prediction model contains only binary or categorical predictors, a discrete distribution may be more appropriate, whereas for skewed or more flexible shapes, a beta or gamma distribution may be preferable. Advice for obtaining the *LP* distribution is given in Section 2.3 and the supplementary material.

We recognize that what is “precise” is subjective. Our examples in Section 3.2 gave suggestions for the O/E, calibration slope and C-statistic based on particular 95% CI widths. The simulation-based calculation identifies the sample size that is expected to give (ie, on average) CIs of the desired width. An alternative is to identify the sample size that gives CIs that are no wider than the desired width on, say, 95% of simulations. This would be even more reassuring but requires even larger sample sizes.

In summary, we propose that precise performance estimates should be targeted when planning external validation studies, and a tailored sample size can be determined through simulation by specifying the likely distribution of the *LP*, the outcome event proportion and target precision for each performance measure. The sample size that, on average, gives the target precision for all performance measures should be selected for the external validation data.

## Appendix Supplementary materials

Image, application 1
