# Supplementary material for: External validation of clinical prediction models: simulation-based sample size calculations were more reliable than rules-of-thumb
Source: J Clin Epidemiol. 2021 Jul;135:79–89. doi: 10.1016/j.jclinepi.2021.02.011 (PMC8352630; doi:10.1016/j.jclinepi.2021.02.011)
Supplement: Supplementary file 1 [file mmc1.docx]

Supplementary Material

**Deciding precision for calibration-in-the-large, O/E and calibration slope**

What is considered ‘precise’ is subjective and the width of the 95% CI for calibration-in-the-large in particular will also depend on the expected outcome proportion. Assuming no miscalibration, the mean *LP* (*μ*) can be selected to give approximately the desired outcome proportion, e.g. outcome proportion ≈ 1/(1+exp(-*μ*) and a sensible CI width could also be based on the probability scale. For example, a 95% CI width of 0.5 for calibration-in-the-large would correspond to a CI width of 0.024 around a probability of 0.05 or width of 0.10 around a probability of 0.3. For the calibration slope, ideally, we would aim for a CI width < 0.2, however this may not be practical in settings with narrow *LP* distributions, as seen in Figure 3, therefore a width of < 0.4 may be more realistic in such settings.

O/E is related to calibration-in-the-large but has a point estimate that is somewhat more interpretable. However, it also depends on the baseline risk (outcome event proportion). With small numbers of events, 95% CIs are wide (95% CI widths > 0.25 with ≤ 100 events). Estimates are also less precise for rarer outcomes as seen in Figure S1 A & B, a the CI width does not appear to depend importantly on the spread of the *LP* (Figure S1 C & D).


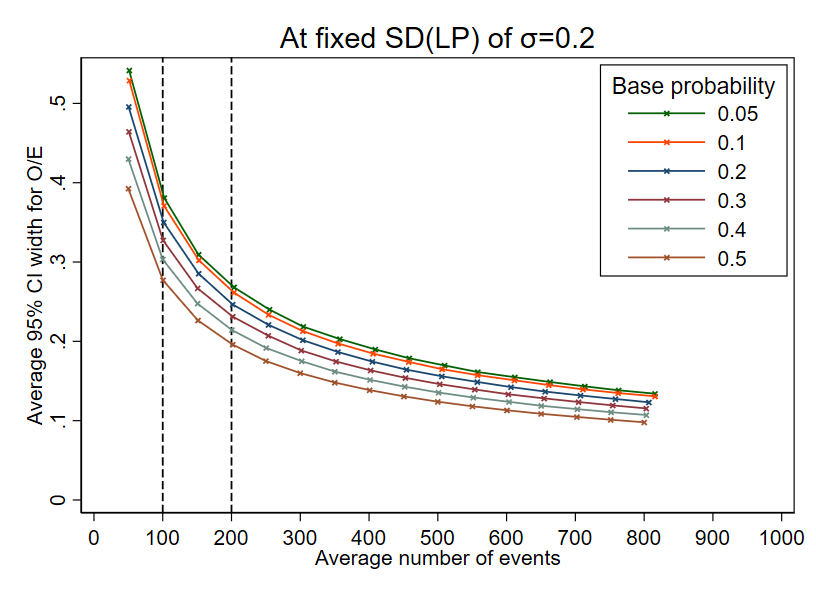

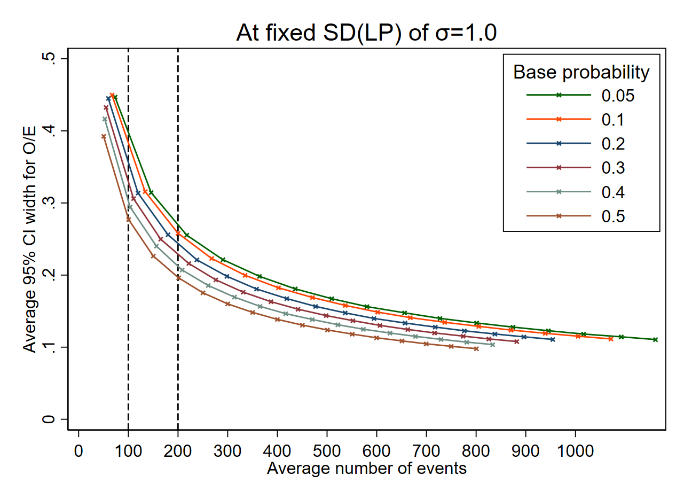

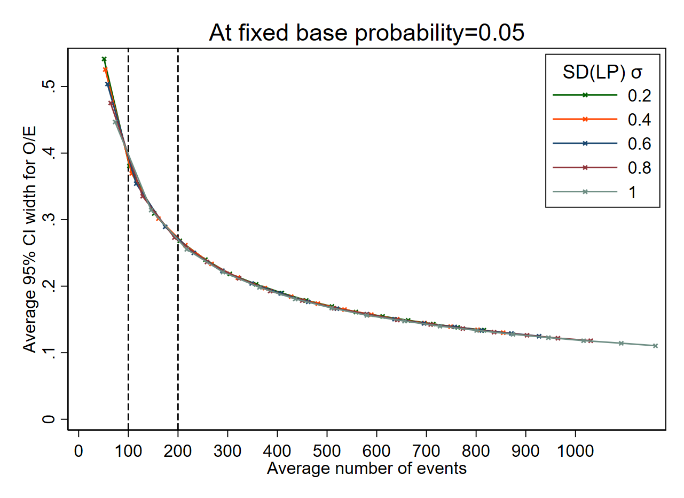

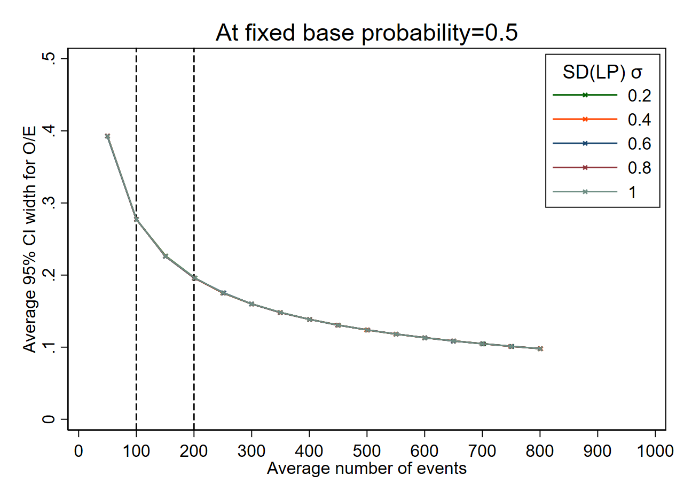


B

C

D

A

Figure S1: Average 95% confidence interval width for the ratio of observed to expected outcome events at different effective sample sizes (based on number of events) comparing by base probabilities at fixed SD(*LP*) (A and B), or comparing by SD(*LP*) at fixed base probabilities (C and D).

Table S1: Average event proportion and SD(*LP*) across simulated datasets with different degrees of miscalibration, compared to the original defining parameters for the scenarios.

| **Scenario defining parameters** | | **Observed mean from simulated datasets** | | | | | | | | |
| --- | --- | --- | --- | --- | --- | --- | --- | --- | --- | --- |
|  |  | **No miscalibration** | **Miscalibration factor = 0.8** | | **Miscalibration factor = 0.9** | | **Miscalibration factor = 1.1** | | **Miscalibration factor = 1.2** | |
| **Base probability** | **SD(*LP*)** | **Event proportion** | **Event  proportion** | **SD(*LP_miscal_*)** | **Event  proportion** | **SD(*LP_miscal_*)** | **Event  proportion** | **SD(*LP_miscal_*)** | **Event  proportion** | **SD(*LP_miscal_*)** |
| 0.05 | 0.20 | 0.05 | 0.09 | 0.16 | 0.07 | 0.18 | 0.04 | 0.22 | 0.03 | 0.24 |
| 0.05 | 0.40 | 0.05 | 0.09 | 0.32 | 0.07 | 0.36 | 0.04 | 0.44 | 0.03 | 0.48 |
| 0.05 | 0.60 | 0.06 | 0.09 | 0.48 | 0.07 | 0.54 | 0.05 | 0.66 | 0.04 | 0.72 |
| 0.05 | 0.80 | 0.06 | 0.10 | 0.64 | 0.08 | 0.72 | 0.05 | 0.88 | 0.04 | 0.96 |
| 0.05 | 1.00 | 0.07 | 0.11 | 0.80 | 0.09 | 0.90 | 0.06 | 1.10 | 0.05 | 1.20 |
| 0.10 | 0.20 | 0.10 | 0.15 | 0.16 | 0.12 | 0.18 | 0.08 | 0.22 | 0.07 | 0.24 |
| 0.10 | 0.40 | 0.11 | 0.15 | 0.32 | 0.13 | 0.36 | 0.09 | 0.44 | 0.07 | 0.48 |
| 0.10 | 0.60 | 0.11 | 0.16 | 0.48 | 0.13 | 0.54 | 0.10 | 0.66 | 0.08 | 0.72 |
| 0.10 | 0.80 | 0.12 | 0.16 | 0.64 | 0.14 | 0.72 | 0.11 | 0.88 | 0.09 | 0.96 |
| 0.10 | 1.00 | 0.13 | 0.17 | 0.80 | 0.15 | 0.90 | 0.12 | 1.10 | 0.11 | 1.20 |
| 0.20 | 0.20 | 0.20 | 0.25 | 0.16 | 0.23 | 0.18 | 0.18 | 0.22 | 0.16 | 0.24 |
| 0.20 | 0.40 | 0.21 | 0.25 | 0.32 | 0.23 | 0.36 | 0.19 | 0.44 | 0.17 | 0.48 |
| 0.20 | 0.60 | 0.22 | 0.26 | 0.48 | 0.24 | 0.54 | 0.20 | 0.66 | 0.18 | 0.72 |
| 0.20 | 0.80 | 0.23 | 0.26 | 0.64 | 0.24 | 0.72 | 0.21 | 0.88 | 0.19 | 0.96 |
| 0.20 | 1.00 | 0.24 | 0.27 | 0.80 | 0.26 | 0.90 | 0.22 | 1.10 | 0.21 | 1.20 |
| 0.30 | 0.20 | 0.30 | 0.34 | 0.16 | 0.32 | 0.18 | 0.29 | 0.22 | 0.27 | 0.24 |
| 0.30 | 0.40 | 0.31 | 0.34 | 0.32 | 0.32 | 0.36 | 0.29 | 0.44 | 0.27 | 0.48 |
| 0.30 | 0.60 | 0.31 | 0.34 | 0.48 | 0.33 | 0.54 | 0.30 | 0.66 | 0.29 | 0.72 |
| 0.30 | 0.80 | 0.32 | 0.35 | 0.64 | 0.33 | 0.72 | 0.31 | 0.88 | 0.30 | 0.96 |
| 0.30 | 1.00 | 0.33 | 0.35 | 0.80 | 0.34 | 0.90 | 0.32 | 1.10 | 0.31 | 1.20 |
| 0.40 | 0.20 | 0.40 | 0.42 | 0.16 | 0.41 | 0.18 | 0.39 | 0.22 | 0.38 | 0.24 |
| 0.40 | 0.40 | 0.40 | 0.42 | 0.32 | 0.41 | 0.36 | 0.40 | 0.44 | 0.39 | 0.48 |
| 0.40 | 0.60 | 0.41 | 0.42 | 0.48 | 0.41 | 0.54 | 0.40 | 0.66 | 0.39 | 0.72 |
| 0.40 | 0.80 | 0.41 | 0.43 | 0.64 | 0.42 | 0.72 | 0.41 | 0.88 | 0.40 | 0.96 |
| 0.40 | 1.00 | 0.42 | 0.43 | 0.80 | 0.42 | 0.90 | 0.41 | 1.10 | 0.40 | 1.20 |
| 0.50 | 0.20 | 0.50 | 0.50 | 0.16 | 0.50 | 0.18 | 0.50 | 0.22 | 0.50 | 0.24 |
| 0.50 | 0.40 | 0.50 | 0.50 | 0.32 | 0.50 | 0.36 | 0.50 | 0.44 | 0.50 | 0.48 |
| 0.50 | 0.60 | 0.50 | 0.50 | 0.48 | 0.50 | 0.54 | 0.50 | 0.66 | 0.50 | 0.71 |
| 0.50 | 0.80 | 0.50 | 0.50 | 0.64 | 0.50 | 0.72 | 0.50 | 0.88 | 0.50 | 0.97 |
| 0.50 | 1.00 | 0.50 | 0.50 | 0.79 | 0.50 | 0.90 | 0.50 | 1.10 | 0.50 | 1.20 |

**Miscalibration**

Figure S2 shows the 95% CI width for the C-statistic plotted against observed and expected numbers of events, where the expected number of events is based on no miscalibration and therefore corresponds to the original simulation scenarios. Panel A, shows no obvious change in precision of the C-statistic due to miscalibration when considering the actual number of events in the datasets, however panel B demonstrates the difference in precision if only the expected number of events is considered. The actual number of events differs to the expected number because of miscalibration.

The findings for other performance statistics were similar in that the precision was not greatly affected when plotted against observed events, but the difference in precision due to miscalibration when plotted against the expected number of events was more pronounced for rare outcome settings (as there is a larger difference between observed and expected numbers of events due to the miscalibration, Table S1). For example, when the base probability is 0.05 and sample size is 6000, the expected number of events is 300 if there is no miscalibration, however the observed number of events ranges from 175 to 523 when there is miscalibration introduced.

B

A

Figure S2: Average 95% confidence interval width for the C-statistic from evaluating a model that is miscalibrated to the data (no miscalibration when miscalibration factor = 1), plotted against the average observed number of events (A) and expected number of events based on the setting without miscalibration (B).

**Obtaining the distribution of the linear predictor from previous studies**

If the validation is being planned by the same researchers that developed the model, they will have access to the development data so can easily summarise the *LP* distribution. However, it will be more challenging if the external validation is planned by an independent research team that do not have access to the development data. When the development data is not available, nor a summary of the *LP* from a publication or model developers, it may be inferred from the distribution of predicted probabilities. For example, calibration plots sometimes include a histogram or dot plot of predicted risks along the x-axis. The approximate median and range of probabilities could be transformed back to the *LP* scale to decide on an appropriate distribution and parameters to assume for simulation.

Also, the supplementary material of Debray et al.[1] shows how the standard deviation of the *LP* is related to the C-statistic, Somer’s D statistic, or Royston’s D statistic, assuming the *LP* is normally distributed. This builds on previous work [2, 3]. Hence, if these discrimination measures are reported, it is possible to obtained the standard deviation of the *LP* indirectly.

Sometimes the *LP* distribution may be available for each outcome group separately (i.e. those with and without an event). Let there be *n* events and *m* non-events, and let $\bar{x}$ and $\bar{y}$ be the means of the *LP* in each of these groups. Then, the mean of the *LP* on in the entire population can be obtained by

$$\bar{LP}=\mu=\frac{n\bar{x}+m\bar{y}}{n+m}$$

Also, let $s_{x}^{2}$ and $s_{y}^{2}$ be the reported unbiased sample variances for each group. Then the variance of *LP* in the population is:

$$\mathrm{var}\left( LP \right)=\sigma^{2}=\frac{\left( n-1 \right)s_{x}^{2}+(m-1)s_{y}^{2}}{n+m-1}+\frac{nm{(\bar{x}-\bar{y})}^{2}}{(n+m)(n+m-1)}$$

as reported here: <https://math.stackexchange.com/questions/2971315/how-do-i-combine-standard-deviations-of-two-groups>

If the validation population is considered different from the development population (e.g. due to change in expected outcome proportion and/or case-mix), a pilot study may be necessary to gauge the distribution better. Or data could be simulated conditional on the assumed multivariate distribution of the values of model predictors in the validation sample, and then the mean and standard deviation calculated.

If an external dataset is already available (i.e. sample size is fixed), the approach can be used to ascertain the expected precision for that particular sample size and observed linear predictor distribution (to help justify its suitability).

[1] Debray TPA, Damen JAAG, Riley RD, Snell KIE, J.B. R, Hooft L, et al. A framework for meta-analysis of prediction model studies with binary and time-to-event outcomes. Stat Methods Med Res. 2018 (in-press).

[2] Austin PC, Steyerberg EW. Interpreting the concordance statistic of a logistic regression model: relation to the variance and odds ratio of a continuous explanatory variable. BMC Med Res Methodol. 2012;12:82.

[3] White IR, Rapsomaniki E, Emerging Risk Factors C. Covariate-adjusted measures of discrimination for survival data. Biom J. 2015;57:592-613.

**Example Stata Code**

The following defines the code the user needs to specify to implement the sample size program (called val_ss), and explains each term. The underlying program is available on request, and will be released as a Stata package in due course.

program val_ss , LPMean(real) LPSd(numlist >0 max=1) ///

CSDiff(numlist >0 max=1) CITLDiff(real) LNOEDiff(real) CSTATDiff(numlist >0 <1 max=1) ///

ICIDiff(real) NBDiff(real) ///

[CSlope(real 1) CITL(real 0) LNOE(real 0) BEGin(integer 100) ///

baseprob(numlist >0 <1 max=1) rep(integer 100) nosscstat nosscslope nosscitl nosslnoe ///

nossici nossnb DETail timing]

lpmean Mean value for LP assuming a normal distribution

lpsd Standard deviation for LP assuming a normal distribution

csdiff Difference between calibration slope estimate and lower or upper 95% CI bound (half 95% CI width)

citldiff Difference between CITL estimate and lower or upper 95% CI bound (half 95% CI width)

lnoediff Difference between ln(O/E) estimate and lower or upper 95% CI bound (half 95% CI width)

Specified for ln(O/E) rather than O/E as O/E has a skewed distribution

cstatdiff Difference between C-statistic estimate and lower or upper 95% CI bound (half 95% CI width)

ICIDiff Difference between ICI estimate and lower or upper 95% CI bound (half 95% CI width)

NBDiff Difference between net benefit estimate and lower or upper 95% CI bound (half 95% CI width)

Optional:

cslope Value for expected calibration slope (default is 1)

citl Value for expected CITL (default is 0)

lnoe Value for ln(O/E) (default is 0)

begin Starting sample size (default and minimum is 100)

baseprob Base probability that can be used to calculate the intercept (i.e. if different to

that based on the mean LP)

rep Number of repetitions to use for simulation (must be >0)

nosscstat Option to exclude C-statistic in the sample size simulation

nosscslope Option to exclude calibration slope in the sample size simulation

nosscitl Option to exclude CITL in the sample size simulation

nosslnoe Option to exclude Ln(O/E) in the sample size simulation

nossnb Option to exclude net benefit in the sample size simulation

nossici Option to exclude ICI in the sample size simulation

detail Provides extra detail on sample size throughout simulation process

timing Shows the time taken to complete the simulation process

**Debray DVT example**

val_ss, lpm(-1.75) lpsd(1.47) csdiff(0.1) cstatdiff(0.05) lnoediff(0.1) ///

nosscitl nossici nossnb beg(100) rep(100) timing detail

An additional option is being developed, so that the user can obtain the expected precision based on a particular sample size that is specified.
